# The genome sequence of rosebay willowherb *Chamaenerion angustifolium *(L.) Scop., 1771 (syn. *Epilobium angustifolium* L., 1753) (Onagraceae)

**DOI:** 10.12688/wellcomeopenres.21163.1

**Published:** 2024-03-20

**Authors:** Maarten J. M. Christenhusz, Andrew R. Leitch, Ilia J. Leitch, Michael F. Fay, Royal Botanic Gardens Kew Genome Acquisition Lab, Plant Genome Sizing collective, Darwin Tree of Life Barcoding collective, Wellcome Sanger Institute Tree of Life Management, Samples and Laboratory team, Wellcome Sanger Institute Scientific Operations: Sequencing Operations, Wellcome Sanger Institute Tree of Life Core Informatics team, Tree of Life Core Informatics collective, Darwin Tree of Life Consortium

**Affiliations:** 1 Royal Botanic Gardens Kew, Richmond, England, UK; 2 Curtin University, Perth, Western Australia, Australia; 3 Queen Mary University of London, London, England, UK

**Keywords:** Chamaenerion angustifolium, fireweed, genome sequence, chromosomal, Myrtales

## Abstract

We present a genome assembly from an individual *Chamaenerion angustifolium* (fireweed; Tracheophyta; Magnoliopsida; Myrtales; Onagraceae). The genome sequence is 655.9 megabases in span. Most of the assembly is scaffolded into 18 chromosomal pseudomolecules. The mitochondrial and plastid genome assemblies have lengths of 495.18 kilobases and 160.41 kilobases in length, respectively.

## Species taxonomy

Eukaryota; Viridiplantae; Streptophyta; Streptophytina; Embryophyta; Tracheophyta; Euphyllophyta; Spermatophyta; Magnoliopsida; Mesangiospermae; eudicotyledons; Gunneridae; Pentapetalae; rosids; malvids; Myrtales; Onagraceae; Onagroideae; Epilobieae; *Chamaenerion*; *Chamaenerion angustifolium* (L.) Scop., 1771 (NCBI:txid13055).

## Background

This plant species is known under a variety of names. In the UK and Ireland, it is commonly referred to as rosebay willowherb (as its leaves resemble those of some willows) and sometimes locally as bombweed, reflecting its ability to rapidly colonise bomb craters (e.g. after the blitz in the Second World War, Hamilton, 2022). More widely, especially in USA, it is known as fireweed as it is a frequent colonizer of the bare ground following forest fires.

**Figure 1.**
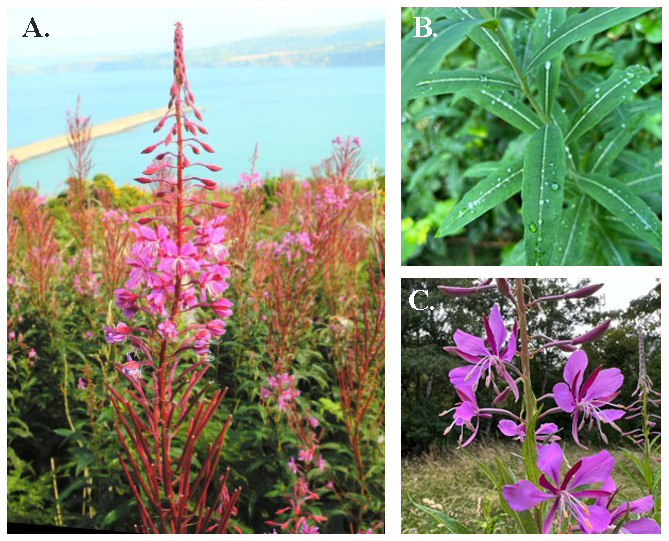
Photographs of *Chamaenerion angustifolium* ( **A**). Plant growing on cliff tops, ( **B**) ( **B**) linear leaves ( **C**) detail of flower, note the reflexed stigma (arrow) and stamens (stars).

The species is a perennial herb, spreading rapidly by underground stolons, and it produces large numbers of hairy wind-dispersed seeds (e.g., some studies estimate a single plant can produce around 80,000 seeds per year, Broderick, 1990), enabling the growth of large, dense stands of tall leafy (up to 2.5 m) stems, topped with spikes of fuchsia-pink flowers ( Figure 1) that are attractive to a large range of insect pollinators, including honeybees. It has a circumpolar distribution, ranging from the arctic/subarctic to cool-temperate regions of North America and Eurasia, to as far south as Mexico, North Africa, the Himalayas, Myanmar and southern China. It is now common and widespread across the UK and Ireland, despite being reported to be rare in the 18th and 19th centuries, when it grew on undisturbed wet, gravelly soils. Its rise in abundance is considered, in part, to reflect its capacity to rapidly colonize and spread in response to changes of land management, an increase in human disturbance and the intensification of (controlled) burning of heathland and forests.

The genus placement of this species has been contentious as it has moved between *Chamaenerion* Ség., *Pyrogennema* Lunell, *Chamerion* (Raf.) Holub. and *Epilobium* L. by various authors. *Chamaenerion* was originally used for all willowherbs (Tournefort, 1700), but Linnaeus preferred the name *Epilobium*, placing *Chamaenerion* into synonymy. Baum *et al.* (1994) found genetic evidence in support of the acceptance of *Chamerion*, which was confirmed by Levin *et al*. (2004), who found the species as sister to the other species of *Epilobium*. There are also some morphological differences such as the alternate leaves, slightly zygomorphic flowers, entire petals and equal length of its eight stamens ( Figure 1C, Wagner *et al.*, 2007), but these do not separate it from all other *Epilobium* species, especially when the deeply embedded former genus *Zauschneria* is included. These also have zygomorphic flowers and frequently alternate leaves. There has also been widespread confusion on the usage of *Chamerion* versus the earlier name *Chamaenerion*, as the latter was originally believed to be illegitimate ( Sennikov, 2011). Here we treat this species as *Chamaenerion angustifolium* as NCBI recognise it as belonging to a separate genus from *Epilobium* (although *Epilobium angustifolium* is listed as a homotypic synonym) and it is the name under which all the genomic data generated here are stored. We note that the name *Epilobium* refers to the position of the petals above the ovary (from Greek *epi*, upon, and *lobos*, lobe), while *Chamaenerion* is derived from Greek *chamai*, low, and *nerion*, oleander. The species name ‘ *angustifolium*’ refers to the narrow leaves of this species.

The species is reported to be used by humans in numerous ways. For example, the pith of the stems can be eaten, and it is considered to be a good additive to soups and stews. It has a flavour of sweet cucumber with a peppery aftertaste ( Kirtley, 2024). Dry stems can be used as twine or to light fires while extracts are used in some creams, shampoos and other cosmetic products, especially for acne ( Adamczak *et al*., 2019). It is also widely used in traditional medicine as it is reported to have anti-cancer, anti-bacterial, anti-inflammatory, and anti-aging properties due to the presence of a diversity of polyphenols and secondary metabolites (reviewed in Prasad *et al.*, 2018).

Variation in the number of chromosomes per cell has been reported between individuals, with counts of 2 *n* = 36, 54, 72 or 108 corresponding to diploid (2 *x*), triploid (3 *x*), tetraploid (4 *x*), and hexaploid (6 *x*) cytotypes, respectively (based on a basic set of 18 chromosomes; i.e. *n* = 18). This variation is due to the occurrence of autopolyploidy ( Husband & Schemske, 1998; Mosquin, 1967), and indeed *C. angustifolium* has been used as a key model species to study the causes and consequence of autopolyploidy in plant evolution and ecology (e.g. Husband, 2004; Husband & Schemske, 1998; Walczyk & Hersch-Green, 2019). Despite this variation, all chromosome counts for material sampled in Britain and Ireland so far have been diploid (i.e. 2 *n* = 2 *x* = 36, (e.g. Henniges *et al.*, 2022; Raven & Moore, 1964).

Here we present the first high-quality genome of *C. angustifolium.* Its genome will not only provide an important baseline resource for studying the evolution of autopolyploids in this model species, but also for helping to dissect the biochemical pathways that lead to the production of over 250 metabolites that may be bioactive and explain the widespread use of this species in traditional medicine ( Adamczak *et al.*, 2019; Kadam *et al.*, 2018).

## Genome sequence report

The genome was sequenced from a specimen of *Chamaenerion angustifolium* ( Figure 1) collected from the Royal Botanic Gardens Kew, Richmond, Surrey, UK (51.48, –0.30). Using flow cytometry, the genome size (1C-value) was estimated to be 0.85 pg, equivalent to 840 Mb. A total of 40-fold coverage in Pacific Biosciences single-molecule HiFi long reads and 64-fold coverage in 10X Genomics read clouds was generated. Primary assembly contigs were scaffolded with chromosome conformation Hi-C data. Manual assembly curation corrected 26 missing joins or mis-joins and removed 2 haplotypic duplications, reducing the assembly length by 0.48%, and increasing the scaffold number by 14.68% and the scaffold N50 by 68.75%.

**Figure 2.**
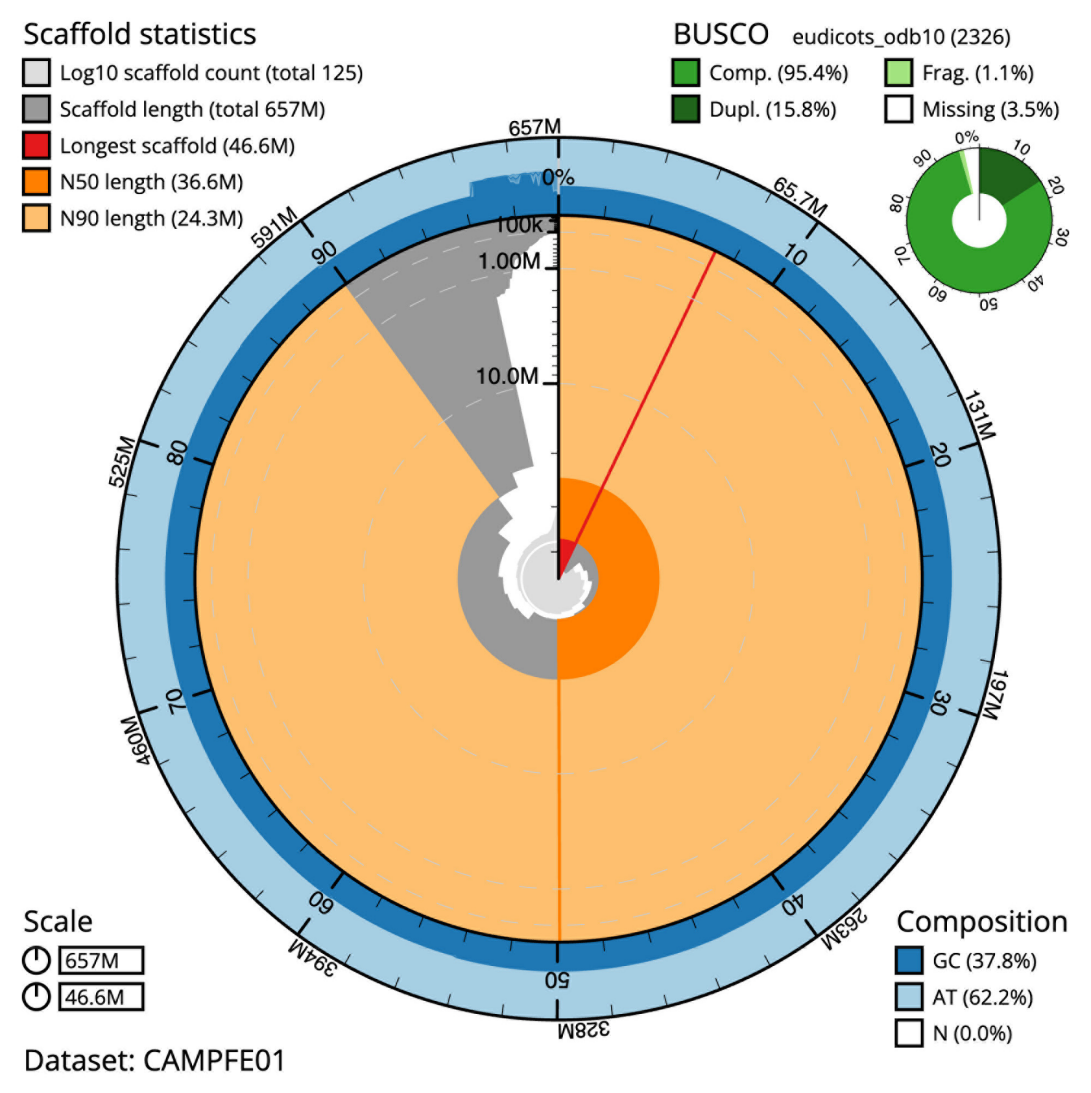
Genome assembly of *Chamaenerion angustifolium*, drChaAngu1.1: metrics. The BlobToolKit snail plot shows N50 metrics and BUSCO gene completeness. The main plot is divided into 1,000 size-ordered bins around the circumference with each bin representing 0.1% of the 656,518,074 bp assembly. The distribution of scaffold lengths is shown in dark grey with the plot radius scaled to the longest scaffold present in the assembly (46,612,786 bp, shown in red). Orange and pale-orange arcs show the N50 and N90 scaffold lengths (36,550,787 and 24,316,059 bp), respectively. The pale grey spiral shows the cumulative scaffold count on a log scale with white scale lines showing successive orders of magnitude. The blue and pale-blue area around the outside of the plot shows the distribution of GC, AT and N percentages in the same bins as the inner plot. A summary of complete, fragmented, duplicated and missing BUSCO genes in the eudicots_odb10 set is shown in the top right. An interactive version of this figure is available at https://blobtoolkit.genomehubs.org/view/CAMPFE01/dataset/CAMPFE01/snail.

**Figure 3.**
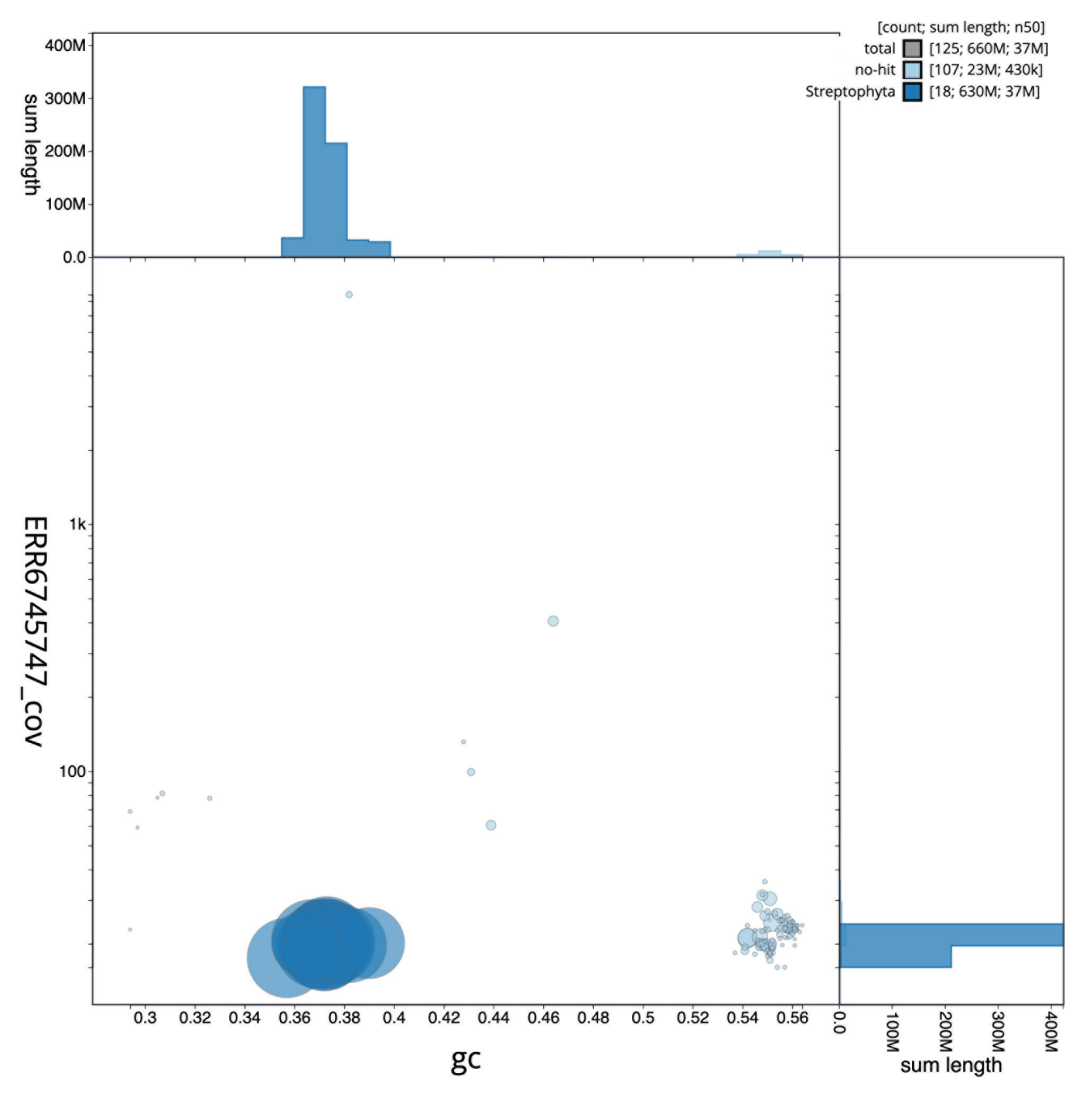
Genome assembly of *Chamaenerion angustifolium*, drChaAngu1.1: BlobToolKit GC-coverage plot. Scaffolds are coloured by phylum. Circles are sized in proportion to scaffold length. Histograms show the distribution of scaffold length sum along each axis. An interactive version of this figure is available at https://blobtoolkit.genomehubs.org/view/CAMPFE01/dataset/CAMPFE01/blob.

**Figure 4.**
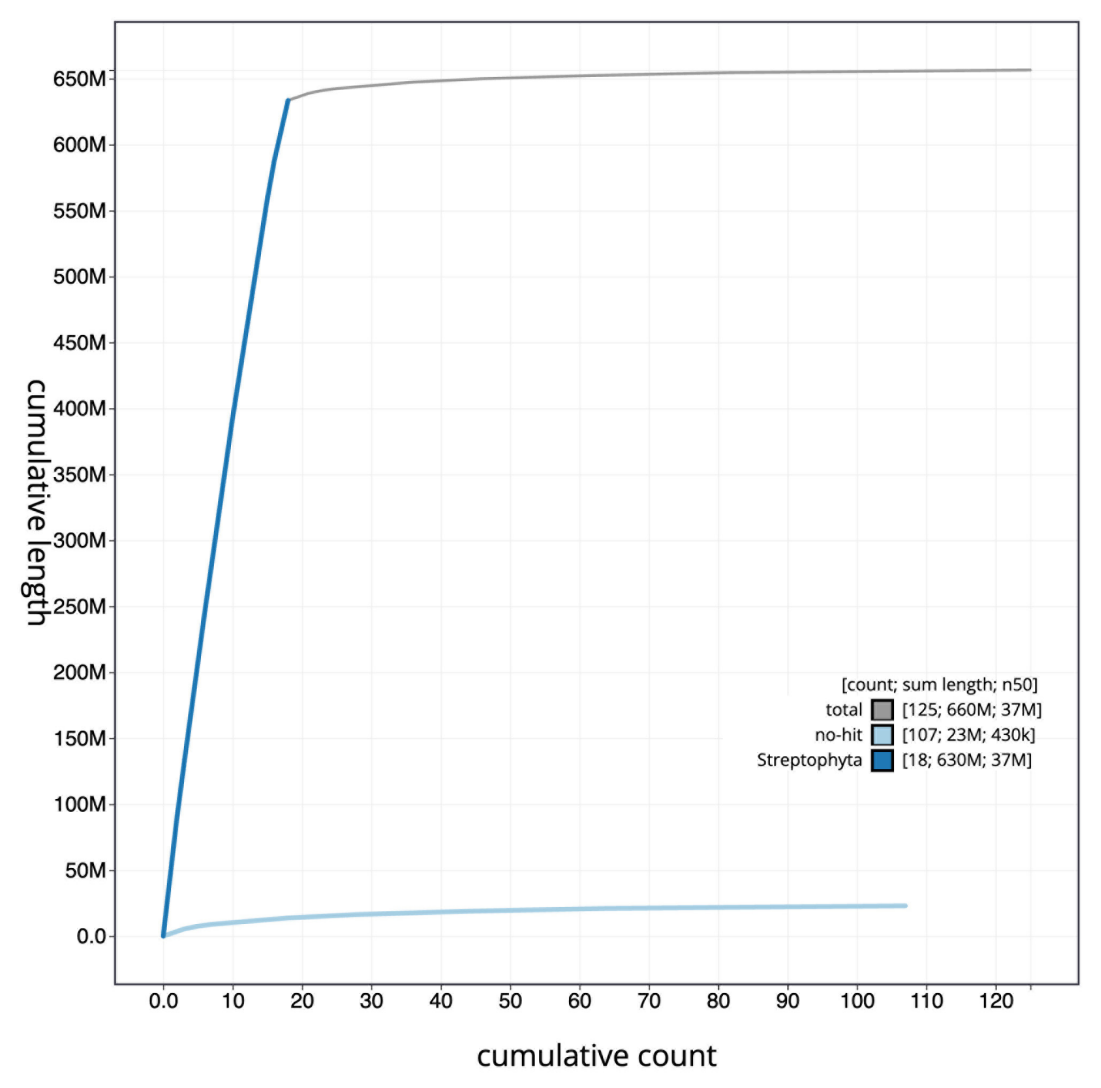
Genome assembly of *Chamaenerion angustifolium*, drChaAngu1.1: BlobToolKit cumulative sequence plot. The grey line shows cumulative length for all scaffolds. Coloured lines show cumulative lengths of scaffolds assigned to each phylum using the buscogenes taxrule. An interactive version of this figure is available at https://blobtoolkit.genomehubs.org/view/CAMPFE01/dataset/CAMPFE01/cumulative.

**Figure 5.**
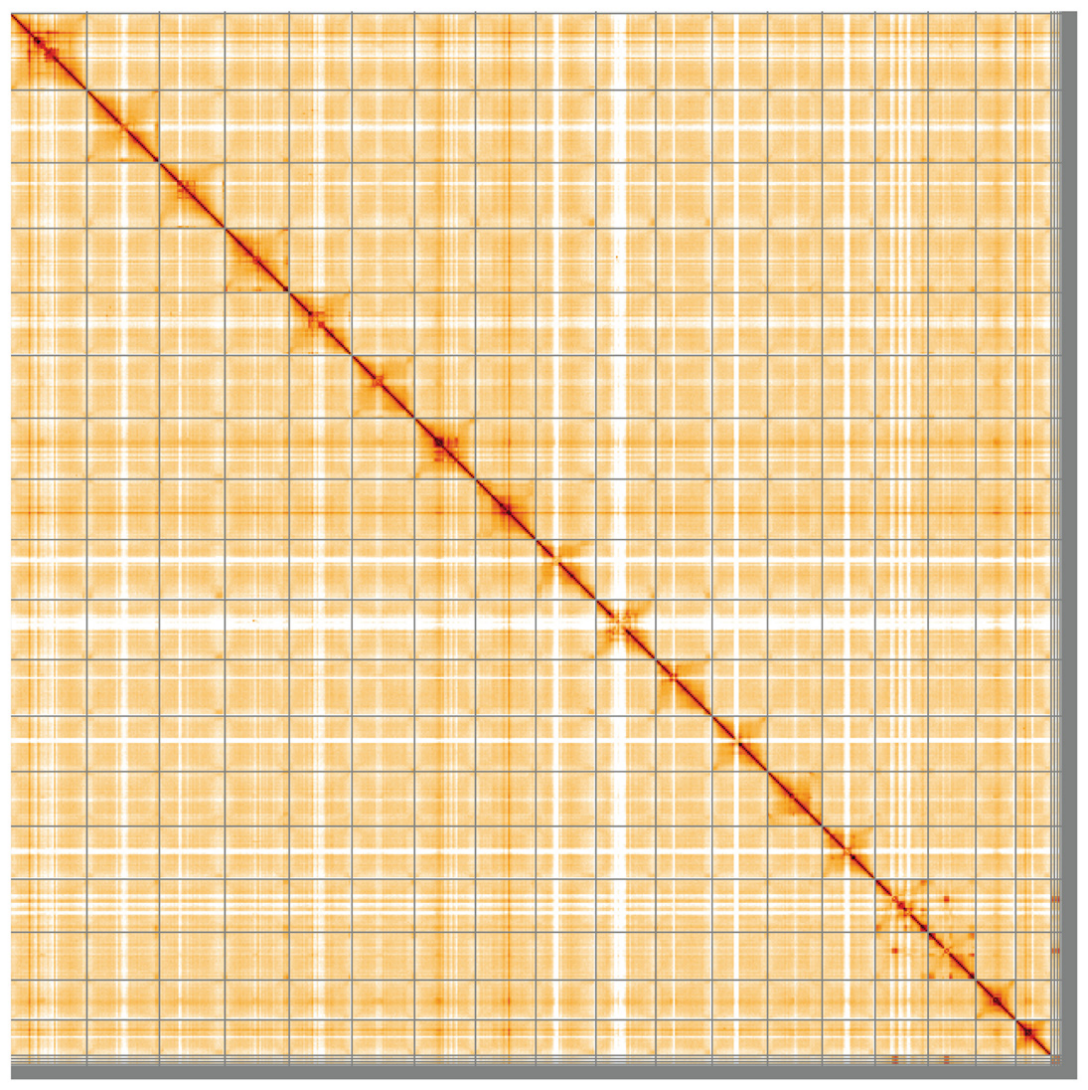
Genome assembly of *Chamaenerion angustifolium*, drChaAngu1.1: Hi-C contact map of the drChaAngu1.1 assembly, visualised using HiGlass. Chromosomes are shown in order of size from left to right and top to bottom. An interactive version of this figure may be viewed at https://genome-note-higlass.tol.sanger.ac.uk/l/?d=GpFNn0GfQgScVp3ROZBHTA.

The final assembly has a total length of 655.9 Mb in 123 sequence scaffolds with a scaffold N50 of 36.6 Mb ( Table 1). The snail plot in Figure 2 provides a summary of the assembly statistics, while the distribution of assembly scaffolds on GC proportion and coverage is shown in Figure 3. The cumulative assembly plot in Figure 4 shows curves for subsets of scaffolds assigned to different phyla. Most (96.59%) of the assembly sequence was assigned to 18 chromosomal-level scaffolds. Chromosome-scale scaffolds confirmed by the Hi-C data are named in order of size ( Figure 5; Table 2). While not fully phased, the assembly deposited is of one haplotype. Contigs corresponding to the second haplotype have also been deposited. The mitochondrial and plastid genomes were also assembled and can be found as contigs within the multifasta file of the genome submission.

**Table 1.** Genome data for *Chamaenerion angustifolium*, drChaAngu1.1.

Project accession data		
Assembly identifier	drChaAngu1.1	
Species	*Chamaenerion angustifolium*	
Specimen	drChaAngu1	
NCBI taxonomy ID	13055	
BioProject	PRJEB47394	
BioSample ID	SAMEA7522629	
Isolate information	drChaAngu1: leaf (DNA, Hi-C and RNA sequencing)	
Assembly metrics *		*Benchmark*
Consensus quality (QV)	52.1	*≥ 50*
*k*-mer completeness	99.98%	*≥ 95%*
BUSCO **	C:95.4%[S:79.6%,D:15.8%],F:1.1%,M:3.5%,n:2,326	*C ≥ 95%*
Percentage of assembly mapped to chromosomes	96.59%	*≥ 95%*
Sex chromosomes	None	*localised homologous pairs*
Organelles	Mitochondrial genome: 495.18 kb Plastid genome: 160.41 kb	*complete single alleles*
Raw data accessions		
PacificBiosciences SEQUEL II	ERR6808068, ERR6939283	
10X Genomics Illumina	ERR6745748, ERR6745745, ERR6745746, ERR6745747	
Hi-C Illumina	ERR6745749	
PolyA RNA-Seq Illumina	ERR9435028	
Genome assembly		
Assembly accession	GCA_946814005.1	
*Accession of alternate haplotype*	GCA_946814875.1	
Span (Mb)	655.9	
Number of contigs	147	
Contig N50 length (Mb)	17.5	
Number of scaffolds	123	
Scaffold N50 length (Mb)	36.6	
Longest scaffold (Mb)	47.27	

* Assembly metric benchmarks are adapted from column VGP-2020 of “Table 1: Proposed standards and metrics for defining genome assembly quality” from Rhie *et al.* (2021). ** BUSCO scores based on the eudicots_odb10 BUSCO set using version 5.3.2. C = complete [S = single copy, D = duplicated], F = fragmented, M = missing, n = number of orthologues in comparison. A full set of BUSCO scores is available at https://blobtoolkit.genomehubs.org/view/CAMPFE01/dataset/CAMPFE01/busco.

**Table 2.** Chromosomal pseudomolecules in the genome assembly of *Chamaenerion angustifolium*, drChaAngu1.

INSDC accession	Chromosome	Length (Mb)	GC%
OX328265.1	1	46.61	37.0
OX328266.1	2	44.15	37.0
OX328267.1	3	39.87	37.5
OX328268.1	4	39.13	37.5
OX328269.1	5	38.16	37.0
OX328270.1	6	38.13	37.0
OX328271.1	7	37.07	36.5
OX328272.1	8	36.74	37.5
OX328273.1	9	36.55	37.0
OX328274.1	10	36.45	35.5
OX328275.1	11	34.32	37.0
OX328276.1	12	33.72	37.5
OX328277.1	13	33.22	37.5
OX328278.1	14	32.24	37.5
OX328279.1	15	32.21	38.0
OX328280.1	16	29.03	39.0
OX328281.1	17	24.32	36.5
OX328282.1	18	21.59	36.5
OX328283.1	MT	0.5	46.5
OX328284.1	Pltd	0.16	38.0

The estimated Quality Value (QV) of the final assembly is 52.1 with *k*-mer completeness of 99.98%, and the assembly has a BUSCO v5.3.2 completeness of 95.4% (single = 79.6%, duplicated = 15.8%), using the eudicots_odb10 reference set ( *n* = 2,326).

Metadata for specimens, barcode results, spectra estimates, sequencing runs, contaminants and pre-curation assembly statistics are given at https://links.tol.sanger.ac.uk/species/13055.

## Methods

### Sample acquisition, genome size estimation and nucleic acid extraction

A specimen of *Chamaenerion angustifolium* (specimen ID KDTOL10109, ToLID drChaAngu1) was collected from the Royal Botanic Gardens Kew, Richmond, Surrey, UK (latitude 51.48, longitude –0.30) on 2020-09-08). The specimen was collected and identified by Maarten Christenhusz (Royal Botanic Gardens Kew), and then preserved by freezing at –80°C.

The genome size was estimated by flow cytometry using the fluorochrome propidium iodide and following the ‘one-step’ method as outlined in Pellicer *et al.* (2021). Specifically for this species, the General Purpose Buffer (GPB) supplemented with 3% PVP and 0.08% (v/v) beta-mercaptoethanol was used for isolation of nuclei ( Loureiro *et al.*, 2007), and the internal calibration standard was *Petroselinum crispum* ‘Champion Moss Curled’ with an assumed 1C-value of 2,200 Mb ( Obermayer *et al.*, 2002).

The workflow for high molecular weight (HMW) DNA extraction at the Wellcome Sanger Institute (WSI) includes a sequence of core procedures: sample preparation; sample homogenisation, DNA extraction, fragmentation, and clean-up. In sample preparation, the drChaAngu1 sample was weighed and dissected on dry ice ( Jay *et al.*, 2023). For sample homogenisation, leaf tissue was cryogenically disrupted using the Covaris cryoPREP ^®^ Automated Dry Pulverizer ( Narváez-Gómez *et al.*, 2023). HMW DNA was extracted using the Automated Plant MagAttract v2 protocol ( Todorovic *et al.*, 2023a). HMW DNA was sheared into an average fragment size of 12–20 kb in a Megaruptor 3 system with speed setting 30 ( Todorovic *et al.*, 2023b). Sheared DNA was purified by solid-phase reversible immobilisation ( Strickland *et al.*, 2023): in brief, the method employs a 1.8X ratio of AMPure PB beads to sample to eliminate shorter fragments and concentrate the DNA. The concentration of the sheared and purified DNA was assessed using a Nanodrop spectrophotometer and Qubit Fluorometer and Qubit dsDNA High Sensitivity Assay kit. Fragment size distribution was evaluated by running the sample on the FemtoPulse system.

RNA was extracted from leaf tissue of drChaAngu1 in the Tree of Life Laboratory at the WSI using the RNA Extraction: Automated MagMax™ *mir*Vana protocol ( do Amaral *et al.*, 2023). The RNA concentration was assessed using a Nanodrop spectrophotometer and a Qubit Fluorometer using the Qubit RNA Broad-Range Assay kit. Analysis of the integrity of the RNA was done using the Agilent RNA 6000 Pico Kit and Eukaryotic Total RNA assay.

Protocols developed by the WSI Tree of Life core laboratory are publicly available on protocols.io ( Denton *et al.*, 2023).

### Sequencing

Pacific Biosciences HiFi circular consensus and 10X Genomics read cloud DNA sequencing libraries were constructed according to the manufacturers’ instructions. Poly(A) RNA-Seq libraries were constructed using the NEB Ultra II RNA Library Prep kit. DNA and RNA sequencing was performed by the Scientific Operations core at the WSI on Pacific Biosciences SEQUEL II (HiFi), Illumina HiSeq 4000 (RNA-Seq) and Illumina NovaSeq 6000 (10X) instruments. Hi-C data were also generated from leaf tissue of drChaAngu1 using the Arima2 kit and sequenced on the Illumina NovaSeq 6000 instrument.

### Genome assembly, curation and evaluation

Assembly was carried out with Hifiasm ( Cheng *et al.*, 2021) and haplotypic duplication was identified and removed with purge_dups ( Guan *et al.*, 2020). One round of polishing was performed by aligning 10X Genomics read data to the assembly with Long Ranger ALIGN, calling variants with FreeBayes ( Garrison & Marth, 2012). The assembly was then scaffolded with Hi-C data ( Rao *et al.*, 2014) using SALSA2 ( Ghurye *et al.*, 2019). The assembly was checked for contamination and corrected using the gEVAL system ( Chow *et al.*, 2016) as described previously ( Howe *et al.*, 2021). Manual curation was performed using gEVAL, HiGlass ( Kerpedjiev *et al.*, 2018) and PretextView ( Harry, 2022). The mitochondrial and chloroplast genomes were assembled using MBG ( Rautiainen & Marschall, 2021) from PacBio HiFi reads mapping to related genomes. A representative circular sequence was selected for each from the graph based on read coverage.

A Hi-C map for the final assembly was produced using bwa-mem2 ( Vasimuddin *et al.*, 2019) in the Cooler file format ( Abdennur & Mirny, 2020). To assess the assembly metrics, the *k*-mer completeness and QV consensus quality values were calculated in Merqury ( Rhie *et al.*, 2020). This work was done using Nextflow ( Di Tommaso *et al.*, 2017) DSL2 pipelines “sanger-tol/readmapping” ( Surana *et al.*, 2023a) and “sanger-tol/genomenote” ( Surana *et al.*, 2023b). The genome was analysed within the BlobToolKit environment ( Challis *et al.*, 2020) and BUSCO scores ( Manni *et al.*, 2021; Simão *et al.*, 2015) were calculated.

Table 3 contains a list of relevant software tool versions and sources.

**Table 3.** Software tools: versions and sources.

Software tool	Version	Source
BlobToolKit	4.0.7	https://github.com/blobtoolkit/blobtoolkit
BUSCO	5.3.2	https://gitlab.com/ezlab/busco
FreeBayes	1.3.1-17-gaa2ace8	https://github.com/freebayes/freebayes
gEVAL	N/A	https://geval.org.uk/
Hifiasm	0.15.3	https://github.com/chhylp123/hifiasm
HiGlass	1.11.6	https://github.com/higlass/higlass
Long Ranger ALIGN	2.2.2	https://support.10xgenomics.com/genome-exome/software/pipelines/latest/advanced/other-pipelines
MBG	-	https://github.com/maickrau/MBG
Merqury	MerquryFK	https://github.com/thegenemyers/MERQURY.FK
PretextView	0.2	https://github.com/wtsi-hpag/PretextView
purge_dups	1.2.3	https://github.com/dfguan/purge_dups
SALSA	2.2	https://github.com/salsa-rs/salsa
sanger-tol/genomenote	v1.0	https://github.com/sanger-tol/genomenote
sanger-tol/readmapping	1.1.0	https://github.com/sanger-tol/readmapping/tree/1.1.0

### Wellcome Sanger Institute – Legal and Governance

The materials that have contributed to this genome note have been supplied by a Darwin Tree of Life Partner. The submission of materials by a Darwin Tree of Life Partner is subject to the **‘Darwin Tree of Life Project Sampling Code of Practice’**, which can be found in full on the Darwin Tree of Life website here. By agreeing with and signing up to the Sampling Code of Practice, the Darwin Tree of Life Partner agrees they will meet the legal and ethical requirements and standards set out within this document in respect of all samples acquired for, and supplied to, the Darwin Tree of Life Project. 

Further, the Wellcome Sanger Institute employs a process whereby due diligence is carried out proportionate to the nature of the materials themselves, and the circumstances under which they have been/are to be collected and provided for use. The purpose of this is to address and mitigate any potential legal and/or ethical implications of receipt and use of the materials as part of the research project, and to ensure that in doing so we align with best practice wherever possible. The overarching areas of consideration are:

• Ethical review of provenance and sourcing of the material

• Legality of collection, transfer and use (national and international) 

Each transfer of samples is further undertaken according to a Research Collaboration Agreement or Material Transfer Agreement entered into by the Darwin Tree of Life Partner, Genome Research Limited (operating as the Wellcome Sanger Institute), and in some circumstances other Darwin Tree of Life collaborators.

## Data Availability

European Nucleotide Archive: *Chamaenerion angustifolium*. Accession number PRJEB47394; https://identifiers.org/ena.embl/PRJEB47394 ( Wellcome Sanger Institute, 2022). The genome sequence is released openly for reuse. The *Chamaenerion angustifolium* genome sequencing initiative is part of the Darwin Tree of Life (DToL) project. All raw sequence data and the assembly have been deposited in INSDC databases. The genome will be annotated using available RNA-Seq data and presented through the Ensembl pipeline at the European Bioinformatics Institute. Raw data and assembly accession identifiers are reported in Table 1.

## Author information

Members of the Royal Botanic Gardens Kew Genome Acquisition Lab are listed here: https://doi.org/10.5281/zenodo.4786680.

Members of the Plant Genome Sizing collective are listed here: https://doi.org/10.5281/zenodo.7994306.

Members of the Darwin Tree of Life Barcoding collective are listed here: https://doi.org/10.5281/zenodo.4893703.

Members of the Wellcome Sanger Institute Tree of Life Management, Samples and Laboratory team are listed here: https://doi.org/10.5281/zenodo.10066175.

Members of Wellcome Sanger Institute Scientific Operations: Sequencing Operations are listed here: https://doi.org/10.5281/zenodo.10043364.

Members of the Wellcome Sanger Institute Tree of Life Core Informatics team are listed here: https://doi.org/10.5281/zenodo.10066637.

Members of the Tree of Life Core Informatics collective are listed here: https://doi.org/10.5281/zenodo.5013541.

Members of the Darwin Tree of Life Consortium are listed here: https://doi.org/10.5281/zenodo.4783558.
