# Supplementary material for: The genome sequence of rosebay willowherb Chamaenerion angustifolium (L.) Scop., 1771 (syn. Epilobium angustifolium L., 1753) (Onagraceae)
Source: Wellcome Open Res. 2024 Mar 20;9:163. [Version 1] doi: 10.12688/wellcomeopenres.21163.1 (PMC11187524; doi:10.12688/wellcomeopenres.21163.1)
Supplement: Peer review history

# Reviewer report: Reviewer response for version 1

**DOI:** 10.21956/wellcomeopenres.23408.r85699

**Published:** 2024-06-19

**Authors:** Samuli Lehtonen

**Affiliations:** 1 Biodiversity Unit, University of Turku, Turku, Finland

The article presents a genome assembly of Chamaenerion angustifolium (Epilobium angustifolium). The rationale for creating the genome assembly is explained and the work is clearly justified. The protocols applied are documented with sufficient details and are appropriate for the purpose. The datasets are clearly presented and fully accessible. 

 Apparently the sequenced specimen is presumed to be diploid on the basis that so far all the chromosome counts for Britain have been diploid for this species, even if variation in chromosome counts have been reported elsewhere. Perhaps this could be specifically mentioned?

 The paper states that "While not fully phased, the assembly deposited is of one haplotype. Contigs corresponding to the second haplotype have also been deposited." This implies that at least some haplotype phasing has been conducted, although it is not explained in the methods. This part could be clarified.

Are sufficient details of methods and materials provided to allow replication by others?

Yes

Is the rationale for creating the dataset(s) clearly described?

Yes

Are the datasets clearly presented in a useable and accessible format?

Yes

Are the protocols appropriate and is the work technically sound?

Yes

Reviewer Expertise:

Phylogenetics, systematics, taxonomy, botany

I confirm that I have read this submission and believe that I have an appropriate level of expertise to confirm that it is of an acceptable scientific standard.

# Reviewer report: Reviewer response for version 1

**DOI:** 10.21956/wellcomeopenres.23408.r80832

**Published:** 2024-06-18

**Authors:** Anze Svara

**Affiliations:** 1 Cornell University, Ithaca, New York, USA

This is a novel and valuable resource for plant research community that will enable future genomic studies of *Chamaenerion *species. Below are my specific remarks to the manuscript:

 Specific remarks

 Please, include a Jellyfish plot on size estimation and heterozygosity, to validate flow-cytometry results.

 Is there a reason why repeat and gene annotation has not been performed?

 Background

1. I suggest mentioning the plant species name in the first paragraph.
2. Second paragraph: Please, break the first long sentence into multiple sentences.
3. “There are also some morphological differences such as the alternate…” Differences between what? Also, break this sentence up, as it is fairly long. Do the same for similar long sentences throughout the manuscript.
4. “especially when the deeply embedded former genus Zauschneria is included”. What is “embedded” referring to? Try to be more specific.
5. “These” and “alternate leaves” What are these two fphrases referring to? Be more specific.
6. “especially for acne” Please, specify what does it do to acne.
7. Could you please explain how k-mer completeness has been calculated?

Genome sequence report

1. “While not fully phased, the assembly deposited is of one haplotype.” From this section, it is unclear how phasing was done and why the chromosomes have not been phased entirely. Can you elaborate on this in the text? Also, does this mean that haplotype 1 contains additional sequences compared to haplome 2? How were falsely duplicated regions filtered?
2. “The mitochondrial and plastid genomes were also…” Add specifications of the plastid genomes.
3. “The estimated Quality Value (QV) …” I suggest reporting data for haplome 2 as well, i.e. each haplome individually. What is the reason for the relatively low QV?

Methods

1. “The genome was analysed within the BlobToolKit environment” What specifically has been analyzed?

Are sufficient details of methods and materials provided to allow replication by others?

Yes

Is the rationale for creating the dataset(s) clearly described?

Partly

Are the datasets clearly presented in a useable and accessible format?

Partly

Are the protocols appropriate and is the work technically sound?

Partly

Reviewer Expertise:

Plant genetics, genomics, fruit crops

I confirm that I have read this submission and believe that I have an appropriate level of expertise to confirm that it is of an acceptable scientific standard, however I have significant reservations, as outlined above.

# Reviewer report: Reviewer response for version 1

**DOI:** 10.21956/wellcomeopenres.23408.r80827

**Published:** 2024-06-18

**Authors:** Parimalan Rangan

**Affiliations:** 1 QAAFI, The University of Queensland (Ringgold ID: 1974), Saint Lucia, Queensland, Australia; 2 ICAR-National Bureau of Plant Genetic Resources, New Delhi, Delhi, India

Following are the inputs that the authors may consider adding to improve their manuscript:

 1. The authors may add a summary statistics of genome assembly in a table comparing the improvement for addition of each method.  For example, they used HiFi for contig assembly, then used 10x, and finally scaffolded using HiC data.  So each additional information from a different platform, how much the assembly was improved.  This will give readers an overview on what methods they can choose. 

 2. Additionally, they can perform BUSCO analysis with Viridiplantae or the nearest taxonomical order to understand the accuracy level.

 3. The quality of the genome assembly can be assessed using LAI tools.  The authors may look into it.    

 4. Structural annotation using appropriate tools might help them to identify the genes present in the genome.

Are sufficient details of methods and materials provided to allow replication by others?

Yes

Is the rationale for creating the dataset(s) clearly described?

Yes

Are the datasets clearly presented in a useable and accessible format?

Yes

Are the protocols appropriate and is the work technically sound?

Yes

Reviewer Expertise:

Genomics, transcriptomics, molecular biology,

I confirm that I have read this submission and believe that I have an appropriate level of expertise to confirm that it is of an acceptable scientific standard.
